# Sex-Specific Linkages Between Taxonomic and Functional Profiles of Tick Gut Microbiomes

**DOI:** 10.3389/fcimb.2019.00298

**Published:** 2019-08-14

**Authors:** Dasiel Obregón, Emilie Bard, David Abrial, Agustín Estrada-Peña, Alejandro Cabezas-Cruz

**Affiliations:** ^1^Center for Nuclear Energy in Agriculture, University of São Paulo, Piracicaba, Brazil; ^2^School of Environmental Sciences, University of Guelph, Guelph, ON, Canada; ^3^EPIA, INRA, VetAgro Sup, Saint Genès Champanelle, France; ^4^Faculty of Veterinary Medicine, University of Zaragoza, Zaragoza, Spain; ^5^UMR BIPAR, INRA, ANSES, Ecole Nationale Vétérinaire d'Alfort, Université Paris-Est, Maisons-Alfort, France

**Keywords:** functional metagenomics, ticks, taxonomic-funtional linkages, microbiota, microbiome, sex-specific differentiation, functional redundancy

## Abstract

Ticks transmit the most diverse array of disease agents and harbor one of the most diverse microbial communities. Major progress has been made in the characterization of the taxonomic profiles of tick microbiota. However, the functional profiles of tick microbiome have been comparatively less studied. In this proof of concept we used state-of-the-art functional metagenomics analytical tools to explore previously reported datasets of bacteria found in male and female *Ixodes ovatus, Ixodes persulcatus*, and *Amblyomma variegatum*. Results showed that both taxonomic and functional profiles have differences between sexes of the same species. KEGG pathway analysis revealed that male and female of the same species had major differences in the abundance of genes involved in different metabolic pathways including vitamin B, amino acids, carbohydrates, nucleotides, and antibiotics among others. Partial reconstruction of metabolic pathways using KEGG enzymes suggests that tick microbiome form a complex metabolic network that may increase microbial community resilience and adaptability. Linkage analysis between taxonomic and functional profiles showed that among the KEGG enzymes with differential abundance in male and female ticks only 12% were present in single bacterial genera. The rest of these enzymes were found in more than two bacterial genera, and 27% of them were found in five up to ten bacterial genera. Comparison of bacterial genera contributing to the differences in the taxonomic and functional profiles of males and females revealed that while a small group of bacteria has a dual-role, most of the bacteria contribute only to functional or taxonomic differentiation between sexes. Results suggest that the different life styles of male and female ticks exert sex-specific evolutionary pressures that act independently on the phenomes (set of phenotypes) and genomes of bacteria in tick gut microbiota. We conclude that functional redundancy is a fundamental property of male and female tick microbiota and propose that functional metagenomics should be combined with taxonomic profiling of microbiota because both analyses are complementary.

## Introduction

Research on host-microbe interactions have brought us to the realization that organisms are colonized by commensal, symbiotic, and pathogenic microorganisms (Byrd et al., 2018; Cani, 2018; Pasolli et al., 2019), ticks are not the exception (Cabezas-Cruz et al., 2018). However, although there is abundant information regarding the taxonomic composition of different host microbiomes, the functional significance of bacterial communities composition and diversity remains largely unknown (Greay et al., 2018; Pasolli et al., 2019). Recent studies have begun to close the information gap between taxonomy and functional profiling in microbiome research, revealing that the distribution of functional groups is strongly influenced environmental conditions by shaping metabolic niches, while the taxonomic composition is weakly affected. Therefore, functional structure and composition within functional groups constitute independent and complementary variation axes (Louca et al., 2016; Heintz-Buschart and Wilmes, 2018). The study of gene function in complex microbial communities, known as functional metagenomics, has been extensively applied to environmental research (Mendes et al., 2014; Jung et al., 2016) and studies in model organisms (Pasolli et al., 2019) enabling higher-resolution descriptions of microbiomes (Pasolli et al., 2019; Zou et al., 2019).

From a functional point of view, it is now irrefutable that host microbiota shape almost every aspect of host biology (Hall et al., 2017; Tang et al., 2018; Canfora et al., 2019). Computational modeling studies suggested that microbiome diversity is strongly influenced by selection acting on microbes, increasing microbial fitness, whereas selection acting on hosts only influences microbiome diversity when there is parental contribution to the microbiomes of offspring (Zeng et al., 2017). Furthermore, a recent meta-analysis of arthropod microbiomes combined taxa clustering with functional profiles and highlighted the presence of conserved functional groups with redundant metabolic capacities. The functional groups were distributed across different classes of proteobacteria suggesting that environmental filtering shapes the structure of arthropod's microbiota (Degli Esposti and Martinez Romero, 2017).

Available reports are conclusive about the high diversity of bacterial taxa harbored by ticks (Cabezas-Cruz et al., 2018), likely as part of the evolutionary strategy of ticks to cope with their complex life cycle and metabolic deficiencies. Recently, Duron et al. (2018) demonstrated that a symbiont of the genus *Francisella*, F-Om, complements a nutritional deficiency of vitamin B in the blood meal of soft ticks *Ornithodoros moubata*. The genome of F-Om exhibits a substantial level of genome reduction and pseudogenization events were identified in around half of protein-coding sequences (Duron et al., 2018). However, the pathways for synthesis of biotin (B7), riboflavin (B2), and folic acid (B9) are conserved and intact in the F-Om genome (Duron et al., 2018). The presence of vitamin B synthesis pathway genes in *Francisella* is fundamental for tick survival and elimination of these bacteria in tick offspring produced anomalies in tick development (Duron et al., 2018). Similarly, despite massive genome reduction in the *Coxiella*-like symbiont of the hard tick *Amblyomma americanum*, the bacterial genome encodes most major vitamin and cofactor biosynthesis pathways, implicating *Coxiella*-like symbiont (CLEAA) as a vitamin provisioning symbiont (Smith et al., 2015). These studies highlight the functional importance of the catalog of genes encoded in tick metagenomes. Considering the microbial diversity of tick microbiota, we hypothesized that functional contributions of tick microbiome could go beyond vitamin B biosynthesis and that unique gene-encoded functions can be harbored by more than one bacteria species or genera.

A holistic view of the functional contribution of tick microbiome is, however, missing, since available analyses offer little insights into the functional profiles of arthropod-associated microbiota. This study is a proof of concept aimed to evaluate the relation between taxonomic and functional differences in male and female ticks of three species, *Ixodes ovatus, Ixodes persulcatus*, and *Amblyomma variegatum*.

## Materials and Methods

### Data Source

We used a metagenomic dataset described previously (Nakao et al., 2013) containing the metagenomes of tick gut (hereafter samples) obtained from ticks of different species, such as *Amblyomma testudinarium, Haemaphysalis formosensis, Haemaphysalis longicornis, I. ovatus, I. persulcatus, Ixodes ricinus*, and *A. variegatum*, collected in the field in different regions. The tick samples were classified as nymph and adult, and these according to sex, then were pooled (+20 ticks per pool) and the DNA corresponding to bacteria/archaea cells was extracted and filtered. Subsequently, shotgun sequencing of the microbiome was performed by pyrosequencing strategy, on a Roche/454 Genome Sequencer FLX Titanium (Roche Applied Science/454 Life Science, Branford, CT), for more detailed information on the sequencing strategy we refer the reader to Nakao et al. (2013).

The raw metagenomic sequences are available in the DNA Data Bank of Japan (DDBJ) (http://www.ddbj.nig.ac.jp), Sequence Read Archive (SRA) under the accession no. DRA000590. For comparative purposes only species represented by adult stages of both sexes were included in the analysis, using only the metagenomes corresponding to adult male (m) and female (f) *A. variegatum* (AVf, DRX001659 and AVm, DRX001660), *I. ovatus* (IOf, DRX001661 and IOm, DRX001662) and *I. persulcatus* (IPf, DRX001663 and IPm, DRX001664).

### Metagenomics Data Preprocessing, Annotation, and Analysis

Single-end DNA sequences were pre-processed and annotated with the web application server Metagenomic Rapid Annotations using Subsystems Technology (MG-RAST), pipeline version 4 (Meyer et al., 2008). Briefly, the raw sequences were processed by quality control (QC) using SolexaQA software package by removing low-quality segments using the “Dynamic Trim” method (Cox et al., 2010), according to the lowest Phred score of 15 and a maximum of 5 bases below the Phred score. In another stage, the artificial replicate sequences formed by sequencing artifacts were removed (Gomez-Alvarez et al., 2009) and the reads passed a screening step to remove sequences with near-exact matches to human genome using Bowtie software (Langmead et al., 2009).

The features (protein coding regions) identification was based on the protein database M5nr which provides non-redundant integration of many protein databases (Wilke et al., 2012). The taxonomic origin of the features was determinate using the RefSeq database (O'Leary et al., 2016). The functional profiles were analyzed according the three data sources that provide hierarchical arrangements of genes and pathways: SEED subsystem (Aziz et al., 2008), KEGG orthologs (Kanehisa and Goto, 2000) and COG database (Tatusov et al., 2000), however we focused our analyses on SEED database. On the annotation parameters we follow the recommendations of Randle-boggis et al. (2016), using the default values in the MG-RAST which maximize sensibility: maximum *e*-value cut-off of 1e-5, and minimum alignment length of 15 bp, hence, the minimal identity cutoff of 60 and 80% were used for the taxonomical and functional profiling, respectively, allowing trade-off between sensitivity and precision. The abundance profiles were determined using the “Best Hit Classification” method.

During the analysis process, features annotated as bacteria domain were filtered (eukaryote and virus sequences removed), and the dataset was normalized based on negative binomial distribution approach, using “DESeq” method (Anders and Huber, 2010) implemented in the MG-RAST Web server. The metagenome dataset resulting from our QC and feature annotation are publicly available in the MG-RAST database under projects number mgp88242 (IO and IP), and mgp88326 (AV).

### Phylogenomic Analysis

Phylogenetic trees were built using NCBI Taxonomy Browser (Benson et al., 2009; Sayers et al., 2009). The taxonomic ID of all genus identified in previous step were collected using the Taxonomy name/id tool. In order to improve graphical readability, the bacteria were then split in six groups according to phylum or class. Phylogenetic trees were built by submitting the taxonomic ID list of each group to the NCBI Common Tree tool. The graphical output was obtained using “ggtree” R package (Yu et al., 2017), providing tools to build custom phylogenetic trees and associated heatmap.

### Statistical Analysis

The Statistical Analysis of Metagenomic Profiles (STAMP) software package (Parks and Beiko, 2010) was used to compare the relative abundance of the taxonomical and functional features, at all organizational levels, between male and female ticks. Analysis considering all the samples were performed using ANOVA test with Tukey-Kramer *post-hoc* method (IC 95%). Two-side Fisher's Exact Test (IC 95%) was used for comparing the relative abundance of bacteria between sexes within each tick species. Multiple test correction was applied in all the analysis, using the Benjamini–Hochberg FDR (false discovery) method. The selection of features included in the charts was made according to the statistical significance of the differences (*q*-corrected <0.05).

## Results

### Data Filtering and Sequence Annotation

To achieve a quality functional annotation, raw sequences were submitted to a completely new analysis workflow. A high proportion of raw sequence data failed to pass the stringent data cleansing process as performed QC pipeline using MG-RAST (Table 1). In the samples of AV, 64.3% of sequences in AVf and 33.6% in AVm were discarded, while in *Ixodes* spp. the highest proportion of eliminated sequences were in male tick samples, with 79.4% in IPm and 62% in IOm, compared to 15.5 and 12.1% in IPf and IOf, respectively. After QC and features annotation, we found differences among the samples from males and females, including but not limited to higher GC content in the microbiome of male ticks of every species to Table 1. The Bacteria domain was predominant in all samples, although a comparatively minor proportion of sequences were annotated in other domains, including Eukaryote, Archaea, and Virus. Only bacterial sequences were included in further analyses.

**Table 1:** Statistical summary of the process of submission, QC, and features annotation in the MG-RAST web server of metagenomic sequences retrieved from tick gut microbiomes.

**Stages**	**Statistical parameter**	**AVf**	**AVm**	**IPf**	**IPm**	**IOf**	**IOm**
Upload	Base pair count (bp)	10 973 277	14 010 027	20 941 861	17 030 492	20 108 476	8 450 898
	Sequences count	42 258	27 582	37 667	35 544	37 136	33 789
	Sequence Length (bp)	260 ± 184	508 ± 81	556 ± 68	479 ± 114	541 ± 73	250 ± 173
	GC proportion (%)	43 ± 7	55 ± 6	31 ± 8	54 ± 8	36 ± 4	47 ± 7
Post QC	Base pair count (bp)	4 888 443	8 729 399	15 985 228	3 002 495	15 920 721	3 833 115
	Sequences count	15 089	18 303	31 826	7 316	32 659	12 786
	Sequence length (bp)	324 ± 198	477 ± 87	502 ± 73	410 ± 148	487 ± 75	300 ± 181
	GC proportion (%)	41 ± 9	54 ± 6	31 ± 8	47 ± 13	36 ± 4	44 ± 9
Annoted	Protein features	8 730	9 294	25 583	6 759	28 676	8 707
	rRNA features	310	37	345	68	275	82
	Bacteria domain (%)*	90.6	82.6	94.6	92.5	97.6	87.4

* *Proportion of features annotated as Bacteria domain, the rest corresponds to Eukaryote, Archaea, and Virus*.

### Sex-Specific Taxonomic Profiles in the Microbiota of AV, IO, and IP

Although included in the study by Nakao et al. (2013), we performed our own bacterial composition analysis workflow. The results of the rarefaction curves indicate that sampling effort in the samples was sufficient to ensure a comprehensive comparison of their microbiota composition (Supplementary Figure 1A). The rarefaction curves and α-diversity analysis also indicate a higher microbial richness in the microbiota of female ticks compared to males of the same species (Supplementary Figure 1B). However, the α-diversity analysis also showed similar values among ticks from the same species, with the higher α-diversity in IO (Supplementary Figure 1B).

The overall bacterial composition of the different samples resulted in 28 phyla, of which Chlamydiae, Proteobacteria, Actinobacteria, and Firmicutes were the four most dominant phyla, accounting for >90% of the bacteria present in the samples (Supplementary Figure 2). However, the distribution of each phylum varied between tick species. The Proteobacteria encompassed >95% of members of the microbial communities of IP, while it only represented 20% in the microbial communities of IO, which were in turn composed mainly (>50%) by representatives of Chlamydiae. In the case of AV, differences were also observed between sexes, wherein Actinobacteria and Proteobacteria were the dominant phyla in AVm and AVf, respectively. The variability of microbial taxa profiles between sex and tick species was also observed analyzing lower taxonomic levels (i.e., family) (Supplementary Figure 2). Furthermore, a pattern of differentiated predominance of related genera in the microbiota according to tick species was observed (i.e., the genera *Actinobacillus, Haemophilus, Lonepinella, Pasteurella, Mannheimia, Aggregatibacter, Histophilus*, and *Basfia*, all belonging to the family *Pasteurellaceae* were mostly present in AV; while the genera *Mycoplasma, Ureaplasma, Mesoplasma, Spiroplasma*, and *Acholeplasma* from the Mollicutes class were predominant in IP) (Supplementary Figure 3).

**Figure 1:**
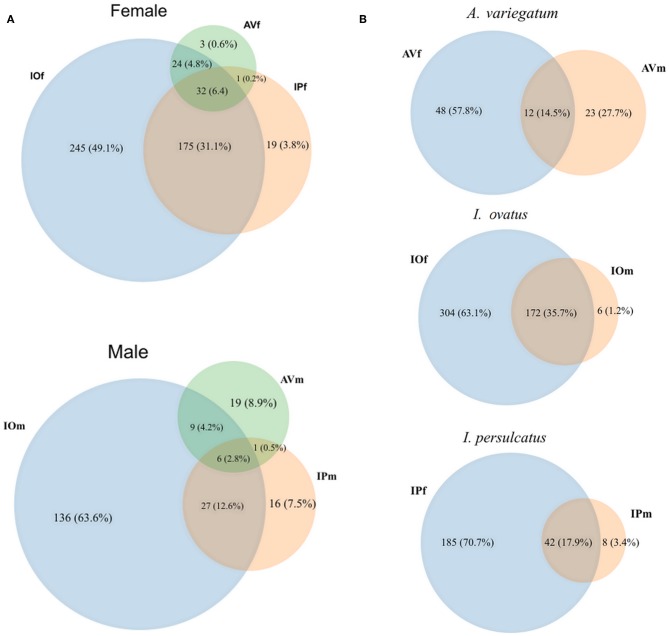
Venn diagrams of bacterial genera composition of the gut microbiota of female and male ticks. **(A)** Comparison of the bacterial genera composition in AV, IP, and IO according to sex. **(B)** Comparisons between female and male in each tick species. The numbers represent the number of genera found in each tick species and those shared by two or all three tick species. The percentage represents their proportionality among the entire set of genera found. Taxonomical features annotated on MG-TAST, based on RefSeq database at minimal identity cut-off of 60%. Graphical representation of Venn diagrams was achieved in BioVinci version 1.1.5 (BioTuring Inc., San Diego, California USA, www.bioturing.com).

**Figure 2:**
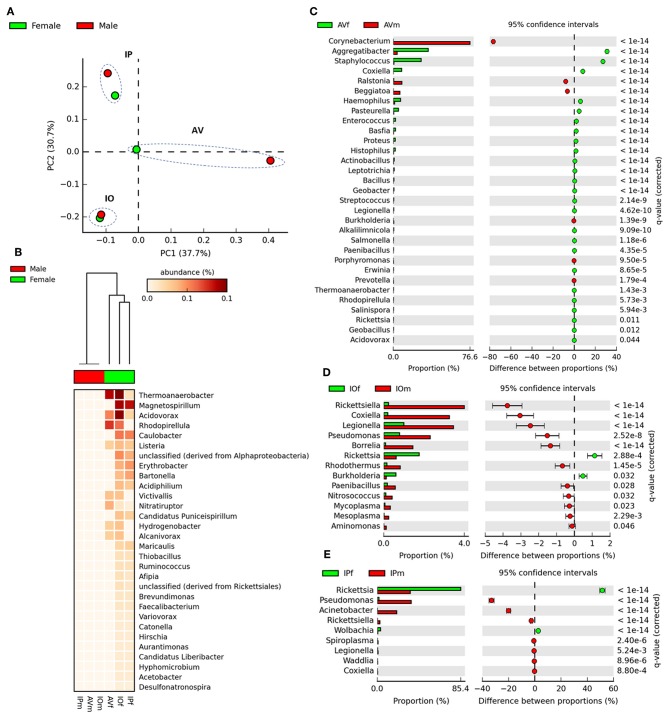
Comparison of taxonomical profiles in the gut microbiota of AVf, AVm, IPf, IPm, IOf, and IOm. **(A)** First axis PCA plot showing the variance of taxonomical profile at level of genera between samples according to tick species and sex, arbitrary ellipses (dashed lines) were drawn to facilitate the interpretation of the figure. **(B)** Heatmap plot showing differences in relative abundance of bacterial genera's in tick gut microbiota between sex. The differences in the abundance of bacterial genera between gut microbiomes from male and female ticks are shown in extended error bars plot for AV **(C)**, IP **(D)**, and IO **(E)**. Only genera with significantly different abundances between males and females are shown (*q* < 0.05).

Venn diagrams of bacterial genera found in the gut microbiota of female and male AV, IP, and IO revealed that the three tick species share only a small proportion of bacterial genera, 6.2% in females and 2.8% in males (Figure 1A). More common bacterial genera were found between *Ixodes* (35.1% in females and 12.6% in males) ticks than between *Ixodes* and *Amblyomma* (5% in females and 4.7% in males). Likewise, the proportion of genera shared between females and males of the same tick species was lower in AV (14.5%) and IP (17.9%) than in IO (35.7%) (Figure 1B). The dissimilarity in gut microbiota composition between tick species was confirmed by principal component analysis (PCA) comparing genera-level abundance profiles in samples from the three tick species and both sexes (Figure 2A). Relative abundance comparison of the microbiota of male and female ticks revealed that several bacterial genera are more abundant in female ticks, regardless of the tick species (Figure 2B). To further explore this clustering; two-side Fisher's exact test was used to compare the relative abundance of bacterial genera in male and female within the same tick species. Results revealed that the abundance of some bacterial genera was significantly different between male and female AV (Figure 2C), IO (Figure 2D), and IP (Figure 2E). The differences in bacterial genera composition between sex were higher in AV than in IO and IP. In addition, the distribution of genera varied between tick species. For example, while AVf has a higher proportion of *Staphylococcus* and *Aggregatibacter*, IOf, and IPf have higher proportion of *Rickettsia* and *Rickettsia* and *Wolbachia*, respectively. IOm, IPm, and AVm have greater abundance of the genera *Rickettsiella, Pseudomonas*, and *Corynebacterium*, respectively.

### Sex-Specific Functional Profiles in the Microbiomes of AV, IO, and IP

The functional metagenomic profiling was based on the SEED database because (i) the functional categories of SEED and KEGG are equivalent and (ii) SEED provides the most consistent and accurate microbial genome annotations of any publicly available source (Meyer et al., 2008; Wilke et al., 2017). Nevertheless, in a first stage, the abundance profiles was also analyzed according to the COG database in order to compare with previous results by Nakao et al. (2013), revealing uniformity in the abundance of functional traits (Supplementary Figures 4A,B). The analysis of functional profiles based on SEED subsystem resulted in 863 functional features (SEED level 4, Supplementary Table 1) identified across all the samples, and these functions were grouped in 26 SEED level 1 categories (Supplementary Figure 4C).

**Figure 3:**
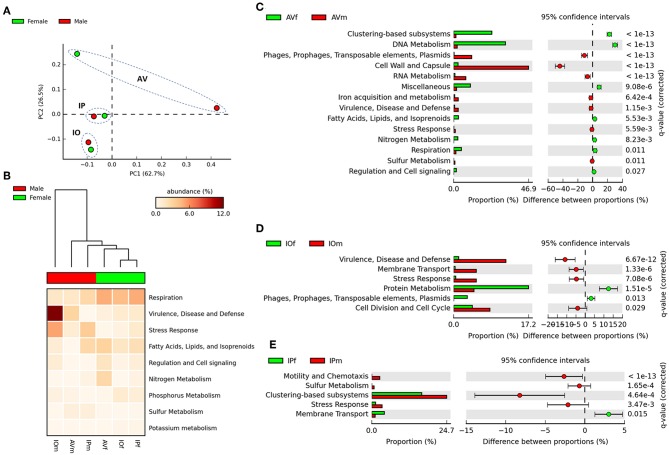
Comparison of functional profiles in the gut microbiota of AVf, AVm, IPf, IPm, IOf, and IOm. **(A)** The first axis PCA plot showing the variance on functional profiling, based on SEED subsystem collapsed at the higher categorical level (level 1), between samples according sex and tick species, arbitrary ellipses (dashed lines) were drawn to facilitate the interpretation of the figure. **(B)** Heatmap plot showing differences between sex of tick on functional traits collapsed at the highest level (SEED, level 1), the functional categories are ranked acordid the effect size, only categories with significant effect in the differences are shown (η^2^ > 0.01). Comparison between sexes is shown as extended error bar plot for AV **(C)**, IP **(D)**, and IO **(E)**. Only the functional categories significantly different in abundances are shown (*q* < 0.05).

In the first stage of the analysis, we explored the similarities between ticks of the same sex, regardless of the tick species. The analysis of similarities ANOSIM based on Bray–Curtis dissimilarity index revealed no differences on averages of rank similarity within groups and between groups (*R* = −0.074; *p* = 0.5). Consequently, no difference (*p* > 0.05) was found for any of the 863 individual functions when the microbiomes of ticks of the same sex were compared (Supplementary Table 1). Then, we observed that similar to taxonomical composition, the PCA on the abundance of functional traits indicated that the sex separated the samples of the same tick species, being the differences between sexes more striking in AV (Figure 3A). Clustering analysis using effect size ANOVA confirmed that the sex influence microbiome composition in functional traits associated with respiration, virulence, and stress response among others (Figure 3B). Therefore, we compared the functional traits of the microbiomes of each tick species, and a detailed analysis of the metabolic profile of these microbiomes revealed significant differences between male and female ticks (Figures 3C–E). In agreement with the PCA analysis, AV have the highest number of categories (i.e., 14) for which significant differences were found between males and females (Figure 3C). A comparatively lower number of categories were found to differentiate the microbiome of males and females of IO (i.e., 6, Figure 3D) and IP (i.e., 5, Figure 3E). Among all the categories only Stress Response was common and showed a significant increase in males of the three species. No other single category was shared in the microbiomes of male and female within each tick species. These results suggest a sex-dependent selection of the microbiome in AV, IO and IP ticks.

### Differences in Carbohydrate and Amino Acid Metabolism Genes in the Microbiomes of Female and Male AV, IO, and IP

**Figure 4:**
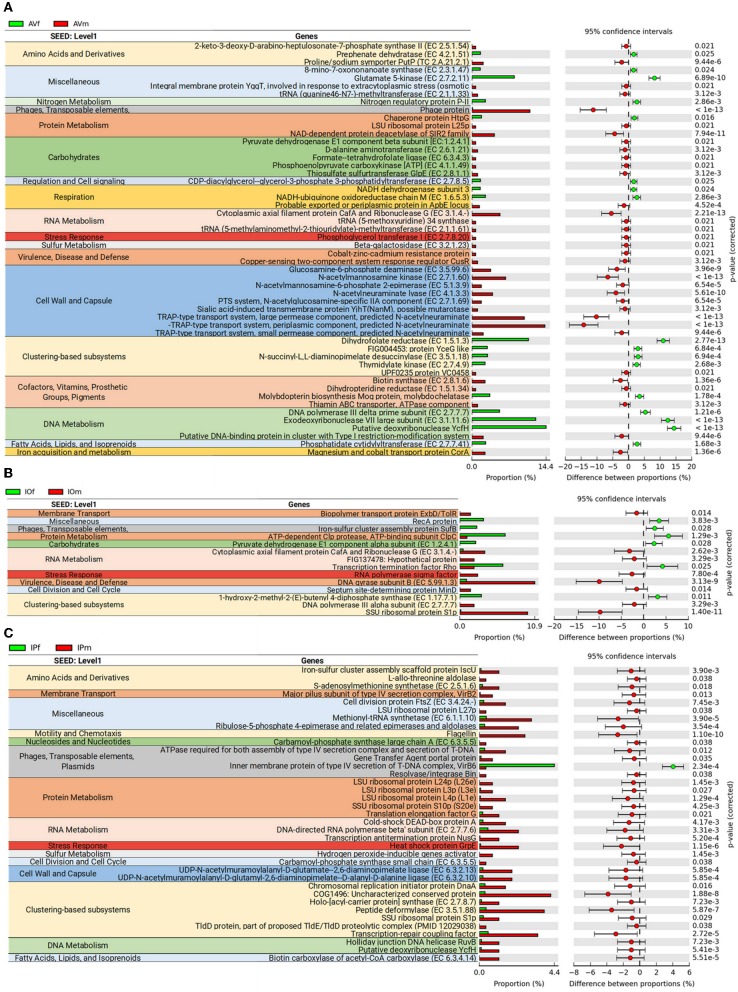
Differences in metabolic functions of tick gut microbiome of AVf, AVm, IPf, IPm, IOf, and IOm. Extended error bar plot showing the more abundant features acording sexes in AV **(A)**, IP **(B)**, and IO **(C)**. Only features with significant differences in relative abundance are shown (*q* < 0.05).

**Figure 5:**
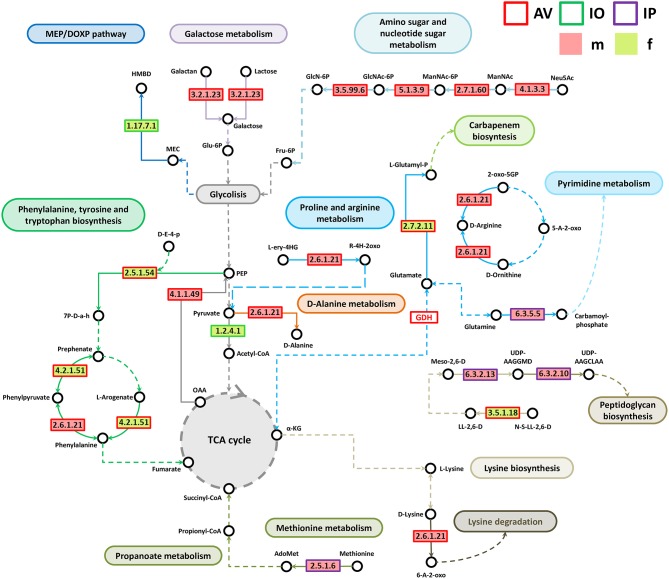
Partial pathway reconstruction of genes involved in amino acid and carbohydrate metabolism. Only metabolic enzymes with significant differences in relative abundance (*q* < 0.05) between male and female ticks and KEGG annotation were included in this analysis. Continuous lines represent reactions catalyzed by the enzymes with significant differences in relative abundance between male and female ticks. Dashed lines represent pathway reconstruction based in KEGG. Fru-6P, Fructose 6-phosphate; GlcNAc-6P, N-acetyl-D-glucosamine 6-phosphate; GlcN-6P, Glucosamine 6-phosphate; Neu5Ac, N-acetylneuraminic acid; ManNAc, N-acetyl-D-mannosamine; ManNAc-6P, N-acetyl-D-mannosamine 6-phosphate; Glu-6P, Glucose-6P; PEP, phosphoenolpyruvate; L-Glutamyl-P, L-glutamate 5-phosphate; HMBPP, (E)-4-hydroxy-3-methyl-but-2-enyl diphosphate; HMBD, 1-hydroxy-2-methyl-2-butenyl 4-diphosphate; MEC, 2-C-Methyl-D-erythritol 2,4-cyclodiphosphate; UDP-AAGGMD, UDP-N-acetylmuramoyl-L-alanyl-gamma-D-glutamyl-meso-2,6-diaminopimelate; UDP-AAGCLAA, UDP-N-acetylmuramoyl-L-alanyl-D-glutamyl-6-carboxy-L-lysyl-D-alanyl-D-alanine; AdoMet, S-adenosylmethionine; a-KG, a-ketoglutarate; L-ery-4HG, L-erythro-4-hydroxy-glutamate; R-4H-2oxo, (R)-4-Hydroxy-2-oxoglutarate; Meso-2,6-D, Mseso-2,6-Diaminopimelate; LL-2,6-D, LL-2,6-Diaminopimelate; N-S-LL-2,6-D, N-Succinyl-LL-2,6-diaminopimelate; 6-A-2-oxo, 6-Amino-2-oxohexanoate; 7P-D-a-h, 7P-2-Dehydro-3-deoxy-D-arabino-heptonate; OAA, Oxaloacetate; GDH, Glutamate dehydrogenase; 5-A-2-oxo, 5-Amino-2-oxopentanoate; 2-oxo-5GP, 2-Oxo-5-guanidino-pentanoate; D-E-4-p, D-Erythose 4-phosphate.

Analysis of bacterial genes composition and abundance between sexes revealed major differences in the metabolism of amino acids, proteins, carbohydrates, respiration, nucleic acids, and chemical elements among others (Figure 4). An analysis of metabolic genes with KEGG annotation shows high representation of genes involved in metabolic interconnections between amino acid and carbohydrate metabolism in the three tick species (Figure 5). Compared to female ticks, AV males show a diverse array of genes involve in the synthesis of metabolic precursors of the glycolysis intermediates fructose-6-phosphate (Fru-6P) and pyruvate. Three key enzymes (EC 4.1.3.3, EC 2.7.1.60, and EC 5.1.3.9) involved in the transformation of N-acetylneuraminic acid (Neu5Ac) in N-acetyl-D-glucosamine 6-phosphate (GlcNAc-6P) were more abundant in AVm than in AVf (Figures 4, 5). The monosaccharide derivative of glucose, GlcNAc-6P, is the main component of the peptidoglycan cell wall in bacteria and can be further metabolized to Fru-6P by two additional enzymatic reactions catalyzed by N-acetylglucosamine-6-phosphate deacetylase (EC 3.5.1.25) and glucosamine-6-phosphate deaminase (EC 3.5.99.6). Of these two enzymes, EC 3.5.99.6 was also most abundant in AVm compared to AVf. The beta-galactosidase (EC 3.2.1.23) that catalyzes the transformation of galactan and lactose in galactose which can then enter glycolysis as glucose-6P (Glu-6P) was also more abundant in AVm. Both metabolites, Glu-6P and Fru-6P, can enter in successive steps of glycolysis to be transformed in phosphoenolpyruvate (PEP) which can be also synthesized from oxaloacetate by the gluconeogenesis enzyme phosphoenolpyruvate carboxykinase (PEPCK, EC 4.1.1.49), another of the enzymes with higher abundance in AVm. Depending on the origin of PEP, catalyzed by PEPCK or enolase, this metabolite has different metabolic fates: rapid transformation to pyruvate, when produced by enolase, or participate in various metabolic processes including gluconeogenesis, glyceroneogenesis, *de novo* serine synthesis pathway, shikimate pathway among others. DAHP synthase (EC 2.5.1.54), highly abundant in AVm, is the first enzyme in the shikimate pathway, which is responsible for the biosynthesis of the amino acids phenylalanine, tyrosine, and tryptophan. DAHP synthase catalyzes the condensation of phosphoenolpyruvate and erythrose 4-phosphate to form 3-deoxy-D-arabino-heptulosonate 7-phosphate and inorganic phosphate. Since DAHP synthase is the first enzyme in the shikimate pathway, it controls the amount of carbon entering the pathway. An additional amino acid aminotransferase (EC 2.6.1.21) involved in phenylalanine, arginine and alanine synthesis, as well as lysine degradation, has higher abundance in AVm.

Only three metabolic enzymes were more abundant in AVf compared with AVm (Figure 4), prephenate dehydratase (EC 4.2.1.51), succinyl-diaminopimelate desuccinylase (EC 3.5.1.18), and glutamate kinase (EC 2.7.2.11). Prephenate dehydratase is involved in two reactions of phenylalanine biosynthesis pathway, the conversion of L-Arogenate and prephenate in phenylalanine and phenylpyruvate, respectively. In contrast to AVm in which one enzyme of lysine degradation pathway was highly abundant, succinyl-diaminopimelate desuccinylase, highly abundant in AVf, is involved lysine biosynthesis. The transformation catalyzed by glutamate kinase, the transfer of a phosphate group to glutamate to form L-glutamate 5-phosphate, is involved in the biosynthesis of proline and, interestingly, the carbapenem antibiotics northienamycin and thienamycin.

The abundance of only two enzymes was significatively different between IOm [(E)-4-hydroxy-3-methylbut-2-enyl-diphosphate synthase, IspG, EC 1.17.7.1] and IOf (pyruvate dehydrogenase, EC 1.2.4.1). IspG catalyzes one of the terminal steps of the MEP/DOXP pathway where it converts 2-C-Methyl-D-erythritol 2,4-cyclodiphosphate in 1-hydroxy-2-methyl-2-butenyl 4-diphosphate which is later transformed in terpenoid backbones. In the mitochondrial matrix, pyruvate is decarboxylated by pyruvate dehydrogenase which is the first step of a series of enzymatic reactions that transform pyruvate into acetyl coenzyme A (Acetyl-CoA).

Four enzymes related with amino acid metabolism were more abundant in IPm than in IPf. In addition to its role in protein synthesis, large amounts of methionine are used for the synthesis of S-adenosylmethionine (AdoMet) by methionine adenosyltransferases (MAT, EC 2.5.1.6) in a reaction that is the rate-limiting step of the methionine cycle. AdoMet participates in a large number of reactions, notably the 5′-deoxyadenosyl moiety of AdoMet participates in biotin synthesis, and thought propanoate metabolism AdoMet can enter to TCA cycle. Other two enzymes, MurE synthetase (EC 6.3.2.13) and MurF synthetase (EC 6.3.2.10), are involve in lysine and peptidoglycan biosynthesis. The first catalyzes the addition of an amino acid to the nucleotide precursor UDP-N-acetylmuramoyl-L-alanyl-D-glutamate (UMAG) in the biosynthesis of bacterial cell-wall peptidoglycan and the second catalyzes the final step in the synthesis of UDP-N-acetylmuramoyl-pentapeptide, the precursor of murein, a polymer that forms a mesh-like layer outside the plasma membrane of most bacteria. The last enzyme (EC 6.3.5.5), carbamoyl-phosphate synthase, uses glutamine to synthesize carbamoyl phosphate, an anion involved in pyrimidines metabolism and arginine biosynthesis.

### Differences in Lipid and Vitamin B Metabolism Genes in the Microbiomes of Female and Male AV, IO, and IP

**Figure 6:**
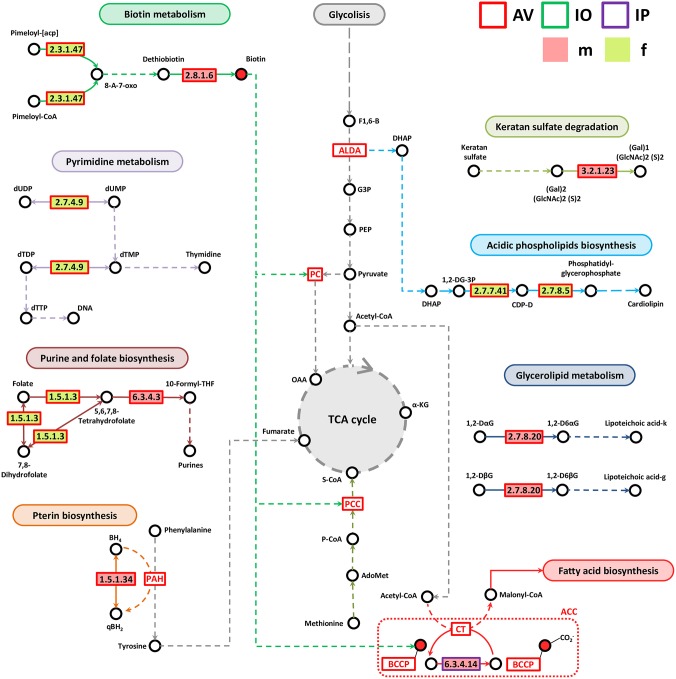
Partial pathway reconstruction of genes involved in lipids, nucleotides and vitamins metabolism. Only metabolic enzymes with significant differences in relative abundance (*q* < 0.05) between male and female ticks and KEGG annotation were included in this analysis. Continuous lines represent reactions catalyzed by the enzymes with significant differences in relative abundance between male and female ticks. Dashed lines represent pathway reconstruction based in KEGG. DHAP, Dihydroxyacetone phosphate; ALDA, Fructose-bisphosphate aldolase; G3P, Glyceraldehyde 3-phosphate; F1,6-B, Fructose 1,6-bisphosphate; 8-A-7-oxo, 8-Amino-7-oxononanoate; PCC, Propionyl-CoA carboxylase; S-CoA, Succinyl-CoA, P-CoA, Propionyl-CoA; LacCer, lactosylceramide; SPH, Sphingomyelin; GlcCer, Glucosylceramide; α-KG, α-ketoglutarate; qBH2, quinoid dihydrobiopterin; PAH, phenylalanine-4-hydroxylase, 1,2-DG-3P, 1,2-Diacyl-sn-glycerol-3P; CDP-D, CDP-Diacylglycerol; 1,2-DaG, 1,2-Diacyl-3-(Glca1-2Glca1)-sn-glycerol; 1,2-DbG, 1,2-Diacyl-3-(Glcb1-6Glcb1)-sn-glycerol; 1,2-D6aG, 1,2-Diacyl-3-(sn-glycerol-1-P-6Glca1-2Glca1)-sn-glycerol; 1,2-D6bG, 1,2-Diacyl-3-(sn-glycerol-1-P-6Glcb1-6Glcb1)-sn-glycerol; Lipoteichoic acid-k, Lipoteichoic acid (kojibiose-containing); Lipoteichoic acid-g, Lipoteichoic acid (gentiobiose-containing); BCCP, biotin carboxyl carrier protein; ACC, Acetyl-CoA carboxylase complex; OAA, Oxaloacetate; PC, pyruvate carboxylase; BH4, Tetrahydrobiopterin; qBH2, quinonoid dihydrobiopterin.

Most of the genes associated with lipid, vitamin B, nucleotide, and Pterin metabolisms were identified in AV (Figure 6). Only one enzyme, biotin carboxylase (EC 6.3.4.14), was found in IP and it was more abundant in IPm compared to IPf (Figure 6). Biotin carboxylase carboxylates the biotin of the biotin carboxyl carrier protein (BCCP) and these two enzymes are part of the acetyl-CoA carboxylase complex (ACC) that catalyzes the irreversible carboxylation of acetyl-CoA to produce malonyl-CoA which provides 2-carbon units to fatty acids and commits them to fatty acid chain synthesis. Two other enzymes involved in biotin synthesis, EC 2.3.1.47 and EC 2.8.1.6, were more abundant in AVf and AVm, respectively. While EC 2.3.1.47 synthesizes the intermediate 8-Amino-7-oxononanoate, biotin synthase (EC 2.8.1.6) catalyzes the final step in the biotin biosynthetic pathway. Biotin plays an important role as a cofactor of several enzymes including pyruvate carboxylase, propionyl-CoA carboxylase and, as mentioned before, ACC (Figure 6).

Enzymes involved in pyrimidine (EC 2.7.4.9) and purine and folate metabolism (EC 1.5.1.3 and EC 6.3.4.3) were more abundant in AVf (EC 2.7.4.9 and EC 1.5.1.3) and AVm (EC 6.3.4.3). Dihydrofolate reductase (EC 1.5.1.3) converts dihydrofolate into folate or tetrahydrofolate which contains methyl groups required for the *de novo* synthesis of purines, thymidylic acid, and certain amino acids. Tetrahydrofolate is then transformed by formate–tetrahydrofolate ligase (EC 6.3.4.3) to 10-formyltetrahydrofolate, a donor of formyl groups in purine biosynthesis and formylation of methionyl-tRNA formyltransferase to give fMet-tRNA.

Pterins are heterocyclic compounds composed of a pteridine ring system with a keto and amino groups. Among them, tetrahydrobiopterin (BH_4_) is an essential cofactor for phenylalanine, tyrosine, and tryptophan hydroxylases. BH_4_ is the product of the dihydropteridine reductase (EC 1.5.1.34), that uses quinonoid dihydrobiopterin (qBH_2_) as substrate, and this enzyme was more abundant in AVm than in AVf. Two other enzymes, EC 3.2.1.23 and EC 2.7.8.20 were also more abundant in AVm than in AVf. The first enzyme EC 3.2.1.23 is a beta-galactosidase that in addition to be involved in galactose synthesis (Figure 5), participates in keratan sulfate degradation (Figure 6). Keratan sulfate is a glycosaminoglycan that plays role as a structural carbohydrate and is widely distributed in the extracellular matrices of animal tissues. The second enzyme EC 2.7.8.20, a phosphoglycerol transferase, may be involved in the synthesis of two glycosyldiacylglycerols (i.e., 1,2-Diacyl-3-(sn-glycerol-1-P-6Glcα1-2Glcα1)-sn-glycerol,1,2-Diacyl-3-(sn-glycerol-1-P-6Glcβ1-6Glcβ1)-sn-glycerol) that may be building blocks for lipoteichoic acid (LTA) synthesis (Figure 6). LTA are major constituents of the cell wall of gram-positive bacteria. Finally, AVf have more abundance of the enzymes phosphatidate cytidylyltransferase (EC 2.7.7.41) and phosphatidylglycerophosphate synthase (EC 2.7.8.5) compared with AVm. These two enzymes catalyze the two final steps in the synthesis of phosphatidyl glycerophosphate that is then transformed into phosphatidylglycerol, a step necessary for the *de novo* biosynthesis of cardiolipin, the major anionic phospholipids in bacteria that functionally interacts with membrane proteins that are involved in energy metabolism and cellular division.

### Linkages Between Taxonomic and Functional Profiles in the Microbiomes of Male and Female AV, IO, and IP

**Figure 7:**
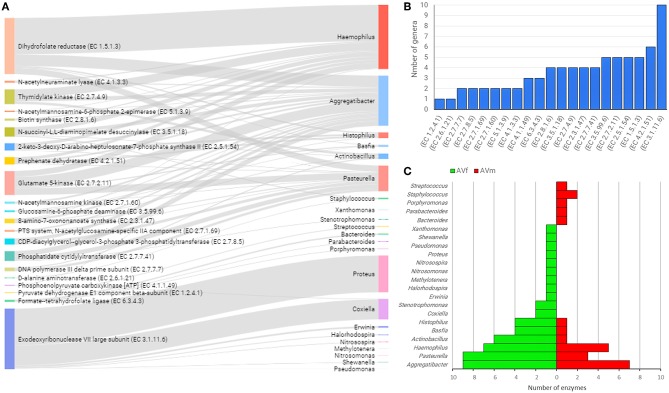
Linkage of enzyme-encoding genes with bacterial genera harboring them into the gut microbiome of AV ticks. Only enzyme-encoding genes with significant differences in relative abundance (*q* < 0.05) between male and female ticks and KEGG annotation were included in this analysis **(A)**. Sankey graph showing the connectivity between functional genes (left) and bacterial genera (right) wherein those were annotated. The edges link genes and source (bacterial genera). The node size is proportional to the number of hits (number of sequences containing the annotation), and consequently the edges size is proportional to the hit's distribution among bacterial genera. **(B)** Bar plot indicating the number of bacterial genera harboring every enzyme-encoding gene. **(C)** Mirror bar plot showing the distribution of enzyme-encoding genes across the bacterial genera in female (AVf) and male (AVm) ticks. A detailed list of enzymes and bacterial source can be found in Supplementary Table 2.

In an attempt to identify possible links between taxonomic and functional profiles, sex-specific functional categories (Figure 4) were linked with the bacterial genera where those genes were annotated. In AV, 23 bacterial genera contain all the genes of interest. Notably, five bacterial genera contained 89.9% of the genes: *Haemophilus* (29.3%)*, Aggregatibacter* (22.8%)*, Proteus* (16.7%)*, Pasteurella* (11.7%), and *Coxiella* (9.4%) (Figure 7A and Supplementary Table 2). Linkage analysis in AV revealed two types of patterns: (i) several enzyme-encoding genes were harbored by the same bacterial genus; (ii) a single enzyme was found in several bacterial genera. About 50% of the genes analyzed in AV were found in four or more bacterial genera. The top enzyme, exodeoxyribonuclease VII large subunit (EC 3.1.11.6), was found in 10 genera (Figure 7B). This enzyme, EC 3.1.11.6, is a bacterial nuclease involved in DNA repair and recombination that hydrolyses single-stranded DNA into large acid-insoluble oligonucleotides, which are then degraded further into small acid-soluble oligonucleotides. Other enzyme-encoding genes widespread among bacterial genera (i.e., found in four or more genera), were associated with amino acids and carbohydrates metabolism (EC 4.2.1.51, EC 2.5.1.54, EC 2.7.2.11, EC 3.5.99.6, and EC 3.5.1.18) and lipid and vitamin B metabolism (EC 2.3.1.47, EC 1.5.1.3, EC 2.7.4.9, EC 2.8.1.6, and EC 2.7.7.41) (Figure 7B). The genera *Aggregatibacter, Pasteurella, Haemophilus, Actinobacillus, Basfia*, and *Histophilus* were found to harbor four or more of the enzyme-encoding genes included in this analysis (Figure 7C). The same genera *Aggregatibacter, Pasteurella, Haemophilus, Actinobacillus, Basfia*, and *Histophilus* contributed to a higher number of enzyme-encoding genes in AVf than in AVm. In addition, other bacterial genera harboring at least one of these genes were found only in AVf or AVm (Figure 7C).

**Figure 8:**
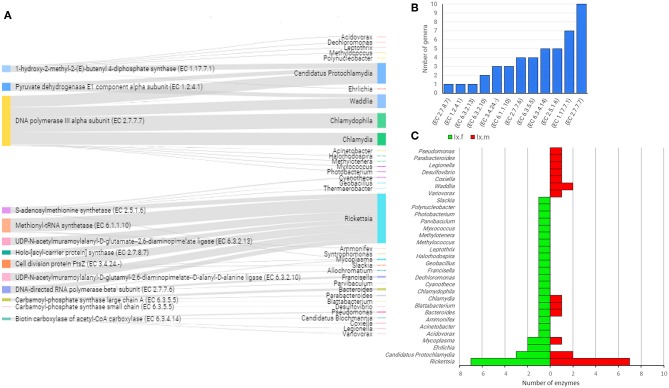
Linkage of enzyme-encoding genes with bacterial genera harboring them into the gut microbiome of both IO and IP ticks. Only enzyme-encoding genes with significant differences in relative abundance (*q* < 0.05) between male and female ticks and KEGG annotation were included in this analysis **(A)**. Sankey graph showing the connectivity between functional genes (left) and bacterial genera (right) wherein those were annotated. The edges link genes and source (bacterial genera). The node size is proportional to the number of hits (number of sequences containing the annotation), and consequently the edges size is proportional to the hit's distribution among bacterial genera. **(B)** Bar plot indicating the number of bacterial genera harboring every enzyme-encoding gene. **(C)** Mirror bar plot showing the distribution of enzyme-encoding genes across the bacterial genera in female (Ix.f) and male (Ix.m) *Ixodes* ticks (data on both ticks species, IO and IP, was merged). A detailed list of enzymes and bacterial source can be found in Supplementary Table 3.

The linkage analysis between the taxonomic and functional profiles in the two *Ixodes* species revealed that the genes of interest were found across 35 bacterial genera and only five genera accounted for 87.8% of hits: *Rickettsia* (39.1%), *Candidatus Protochlamydia* (16.3%), *Chlamydophila* (11.7%), *Waddlia* (10.9%), and *Chlamy*dia (9.8%) (Figure 8A and Supplementary Table 3). The enzyme found in the highest number of genera (i.e., 10) was associated to DNA metabolism, in this case, the enzyme DNA polymerase III (EC 2.7.7.7), which constitute the primary enzyme complex involved in prokaryotic DNA replication. Other enzymes present the genome of four or more bacterial genera include EC 1.17.7.1, EC 2.5.1.6, EC 6.3.4.14, and EC 6.3.5.5 (Figure 8B) for which the function was described above. Differences were also found in the bacterial genera that contributed to the different functional profiles of female and male *Ixodes* ticks (Figure 8C). The genus *Rickettsia* harbored seven enzyme-encoding genes in both female and male ticks, while *Candidatus Protochlamydia* also contributed with three and two enzymes on the microbiome of females and males *Ixodes* ticks, respectively. Nevertheless, 83% (26/31) of the genera contributed with a single functional gene on *Ixodes* tick microbiomes, and this proportion was higher when compared to 56.5% (13/23) in AV.

**Figure 9:**
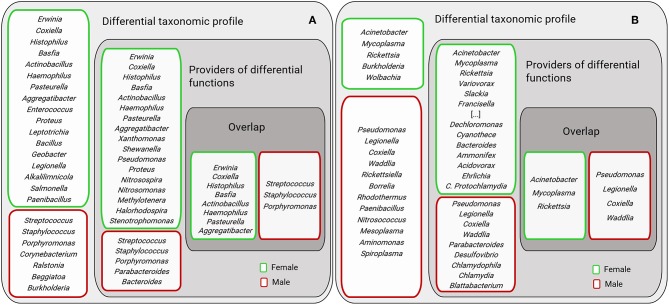
Comparison of bacterial genera identified in the sex-specific taxonomic and functional profiles. The figure displays female and male AV **(A)** and *Ixodes* spp. **(B)**. “Differential taxonomic profiles” include those genera with differential abundance between AVf and AVm (as shown in Figure 2C) and female (Ix.f) and male (Ix.m) *Ixodes* ticks (data on both ticks species, IO and IP, was merged) (as shown in Figures 2D,E). “Providers of differential functions” were described in Figures 7, 8 for AV and Ix, respectively. “Overlap” displays the genera found in both groups “Differential taxonomic profiles” and “Providers of differential functions.” For figure display purposes the following genera were not included: *Polynucleobacter, Photobacterium, Parvibaculum, Myxococcus, Methylotenera, Methylococcus, Leptothrix, Halorhodospira, Geobacillus*, and their position is labeled as “[…]”.

The bacterial genera of the sex-specific taxonomic and functional profiles of the three tick species were compared (Figure 9). Results showed that only a small group of bacterial genera contribute to differentiate males and females metagenomes at both functional and taxonomic levels. Most of the bacterial genera were found to contribute to either the functional or the taxonomic profiles.

## Discussion

The dataset reported by Nakao et al. (2013) is one of the few studies, in addition to Carpi et al. (2011), that used whole-metagenome sequencing to study the taxonomic composition of tick microbiota. Most of the studies are based on the NGS approach using amplicon sequencing of marker genes (Greay et al., 2018). Yet, the functional traits of the microbial communities living in body compartments of ticks, as well as the pattern of the functional diversity remain largely unexplored. The trait-based approaches in microbial ecology research enable a deeper understanding of the role of biodiversity in maintaining multiple ecosystem functioning (Krause et al., 2014; Louca et al., 2016). Such approach has a direct application to tick vectors, for which the debate about the relevance of “*differences in taxonomic profiles only*” is open (Estrada-Peña and Cabezas-Cruz, 2019). Here we used the MG-RAST pipeline (Meyer et al., 2008) version 4 (Meyer et al., 2017) to achieved a workflow substantially different to that followed by Nakao et al. (2013). Using MG-RAST we were able to perform taxonomic and functional feature annotation and profiling of the gut microbiota of males and females of three tick species IO, IP, and AV. Despite the original dataset has the weakness of the reduced number of biological replicates and the limited coverage resulting from a 454 pyrosequencing technology (Liu et al., 2012; Goodwin et al., 2016), the results of this study are meaningful.

The rationale behind our study is rooted on the experiences about the role of *Coxiella* (Smith et al., 2015) and *Francisella* (Duron et al., 2018), proposed to complement some nutritional deficiencies in the tick diet, which was proposed to be one of the functional implications of the tick microbiome (Estrada-Peña and Cabezas-Cruz, 2019). Despite massive genome reduction compared to pathogenic *Francisella* and *Coxiella* species, the genes for biosynthesis of vitamin B were conspicuously intact in the genome of the F-Om and CLEAA symbionts of soft and hard ticks (Smith et al., 2015; Duron et al., 2018). In this study, in addition to bacteria of the genus *Coxiella*, B vitamins biosynthesis genes were also found in *Actinobacillus, Aggregatibacter, Basfia, Ehrlichia, Haemophilus, Histophilus, Neorickettsia, Pasteurella*, and *Rickettsia* in AV, IO, and IP (Figures 6–8). Despite we performed only a partial reconstruction of metabolic pathways and therefore it is not known whether B vitamins biosynthesis pathways are completed; the presence of B vitamins biosynthesis genes in several bacterial genera suggests that bacteria other than *Francisella* and *Coxiella* may also complement the nutritional deficiency of vitamin B in the tick blood meal.

Several other enzyme-encoding genes not related with B vitamins metabolism were also present in four or more bacteria genera of the gut microbiota of AV (Figure 7). The metabolic pathways of these genes were as diverse as amino acid, antibiotics, pyrimidine, lipids, and amino sugars metabolism suggesting that the contribution of tick gut microbiota can go beyond B vitamins supplementation. Highly redundant enzymes (i.e., present in four or more bacteria genera) were also found in *Ixodes* tick microbiomes (Figure 8) and these enzymes are also involved in very disparate pathways including MEP/DOXP, amino acid degradation, DNA replication, fatty acid biosynthesis, and pyrimidines biosynthesis. The presence of the same genes and functions in different bacteria of the microbiota implies functional redundancy, a property of microbiota reported in other species, for example humans (Heintz-Buschart and Wilmes, 2018), but poorly studied in arthropod vectors. Functional redundancy can increase tick resilience in case of perturbations affecting the taxonomic composition of the bacterial community within the tick microbiota (Estrada-Peña et al., 2018). In contrast to the long-term mutualism of *Francisella* with ticks (Duron et al., 2018), the capacity of ticks to harbor a variable bacterial composition according to the life stage and the changing environmental conditions may contribute to tick survival in extreme environments and stressful conditions.

The presence of stress response genes in the metagenome of male, compared to female ticks, of the three species included in this study (Figure 3), suggests that male ticks may maximize the presence of bacteria containing this gene category to cope with specific conditions to which they are exposed. While the sole presence of genes in the metagenome does not guaranty a role in tick physiology, the differences in the life cycle of males and females could help explaining why all males have more stress response genes than females. For example in *Ixodes* spp., they quest for a host, but mate females commonly before or while feeding. The need for a prolonged survival of males even under adverse conditions is even more evident in the case of some *Amblyomma* spp., males being the first stages reaching a suitable host and then emitting a “call pheromone” to stimulate the females to climb on the host already occupied by males (Sonenshine and Mather, 1994). In both cases, male ticks should be better prepared to cope with environmental stress (e.g., exhaustion of hydric resources (Sonenshine and Mather, 1994) than females.

Surprisingly, the bacterial genera providing major functional differences between males and females did not match completely the most abundant bacterial genera in males and females, respectively (Figure 9). We hypothesized that this could result from sex-specific selective pressures that act independently on the phenomes (set of phenotypes) and the genomes of bacteria in the tick gut microbiota. In a simple model, “one genome-one phenome,” the genome of a single bacterium could easily predict its phenome. For example, genome-phenome correspondence analysis of *Burkholderia cenocepacia* during long-term chronic infection identified a number of candidate genes that were highly associated with the motility and biofilm phenotypes of these bacteria (Lee et al., 2017) and the specific inhibition of one of these genes may affect one trait and not the other. However, the simultaneous evolution of hundreds of bacteria within a host allows a playground for evolution where a high number of trait complementation could emerge from trait-by-trait phenomena, including horizontal gene transfer, epistasis (Zeng et al., 2017) and metabolic networks resulting from community-based phenomes. For example, fitness traits of *Drosophila melanogaster* could not be tracked to specific bacteria of the fly microbiota, but instead microbiota bacteria interactions were shown to shape both microbiome abundance and host fitness traits (Gould et al., 2018). In this framework, it can be hypothesized that bacteria in the group “Providers of differential functions” (Figure 9) may be selected based on community-dependent phenomes that are influenced by sexual traits of the ticks. Other bacteria in the group “Differential taxonomic profiles” (Figure 9) may be “independent players” selected for their individual phenome. Finally, bacteria genera in the groups “Overlap” may be selected based on both individual and community-dependent phenomes.

## Conclusion

Functional metagenomics is a powerful analytical tool that complements microbiota composition studies. The high diversity of tick microbiome and the rich functionality of tick metagenomes suggest that these arthropods evolved mechanisms to maximize the genetic diversity of the microorganisms they harbor, which in turn may increase the functional capabilities of the ticks. Hosting highly diverse and redundant microbiomes may offer ticks a competitive advantage in the environment. Differences in taxonomical and functional microbiomes between tick species, and even more between females and males of the same species, suggest the specialization of tick gut microbiota in response to peculiarities of the life cycle and fitness of the tick hosts. In agreement with this hypothesis, a strong phylosymbiotic signal revealed that phylosymbiosis may be a widespread phenomenon in tick-microbiota evolution (Díaz-Sánchez et al., 2019). This finding, together with the results of the current study, supports the existence of a species-specific and sex-specific tick hologenome with a largely unexplored influence on tick biology and pathogen transmission. We proposed that the characterization of tick gut microbiome should be undertaken in terms of functional and taxonomic diversity instead of only taxonomic diversity.

## Data Availability

Publicly available datasets were analyzed in this study. This data can be found here: https://www.nature.com/articles/ismej2012171.

## Author Contributions

AC-C, AE-P, and DO conceived the study. DO, AC-C, EB, and DA analyzed the data. AC-C and DO drafted the manuscript. All authors read the manuscript and made critical revisions.

### Conflict of Interest Statement

The authors declare that the research was conducted in the absence of any commercial or financial relationships that could be construed as a potential conflict of interest.

## Supplementary Material

Supplementary Figure 1 Species richness in the gut microbiota of AVf, AVm, IPf, IPm, IOf, and IOm. **(A)** Rarefaction curve of annotated species represented the total number of distinct species as a function of the number of sequences sampled. **(B)** α-diversity values summarize the species richness in each sample. The species-level annotations were performed in MG-RAST and species richness calculated as the antilog of the Shannon diversity.

Supplementary Figure 2 Phylum-level and Family-level taxonomic profiles of gut microbiota of AVf, AVm, IPf, IPm, IOf, and IOm. Taxonomical features annotated on MG-TAST, based on RefSeq database at minimal identity cutoff of 60%. Only the most abundant phyla are shown in the legend.

Supplementary Figure 3 Phylogenetic tree and relative abundance of bacterial genera found in the gut microbiota of AVf, AVm, IPf, IPm, IOf, and IOm. The relative abundance of each genus is indicated by the scale bar, and bars are pattern-coded for each tick species and sex. Phylogenetic trees were built using NCBI Taxonomy Browser. For this purpose the taxonomic ID of all genus identified in the taxonomic profiling in MG-RAST pipeline (based on RefSeq database at minimal identity cutoff of 60%) were collected using the Taxonomy name/id tool. The bacteria were then split in six groups according to phylum or class, and the phylogenetic trees were built by submitting the taxonomic ID list of each group to the NCBI Common Tree tool.

Supplementary Figure 4 The functional profile of AVf, AVm, IPf, IPm, IOf, and IOm based on COG database. **(A)** Abundance profiles of functions clustered at Level 1 **(B)** and Level 2. Protein features annotated following the MG-RAST pipeline, profiles acording representative hit method, with maximum e-value cut-off of 1e-5, minimal identity cut off of 80%; and minimum alignment length of 15 bp. **(C)** The functional profile of gut microbiome of AVf, AVm, IPf, IPm, IOf, and IOm based on the SEED subsystem database at categorical Level 1, with minimal identity cutoff of 80%.

Supplementary Table 1 Comparison of all the functional features annotated in the metagenomes of female and males AV, IO, and IP. Annotation based on SEED at level 4.

Supplementary Table 2 Linkage list of enzymes and their bacterial sources in AV.

Supplementary Table 3 Linkage list of enzymes and their bacterial sources in IP and IO.
