# Supplementary figures and images for: Sex-Specific Linkages Between Taxonomic and Functional Profiles of Tick Gut Microbiomes
Source: Front Cell Infect Microbiol. 2019 Aug 14;9:298. doi: 10.3389/fcimb.2019.00298 (PMC6702836; doi:10.3389/fcimb.2019.00298)

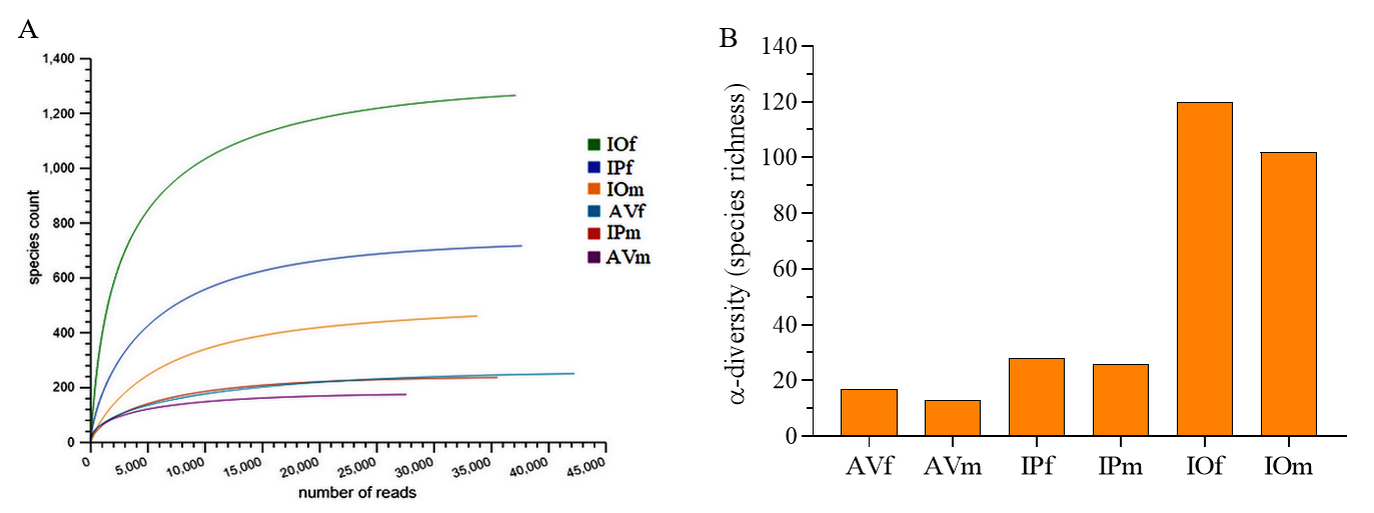

Supplement: Supplementary Figure 1 — Species richness in the gut microbiota of AVf, AVm, IPf, IPm, IOf, and IOm. (A) Rarefaction curve of annotated species represented the total number of distinct species as a function of the number of sequences sampled. (B) α-diversity values summarize the species richness in each sample. The species-level annotations were performed in MG-RAST and species richness calculated as the antilog of the Shannon diversity. [file Image_1.TIF]

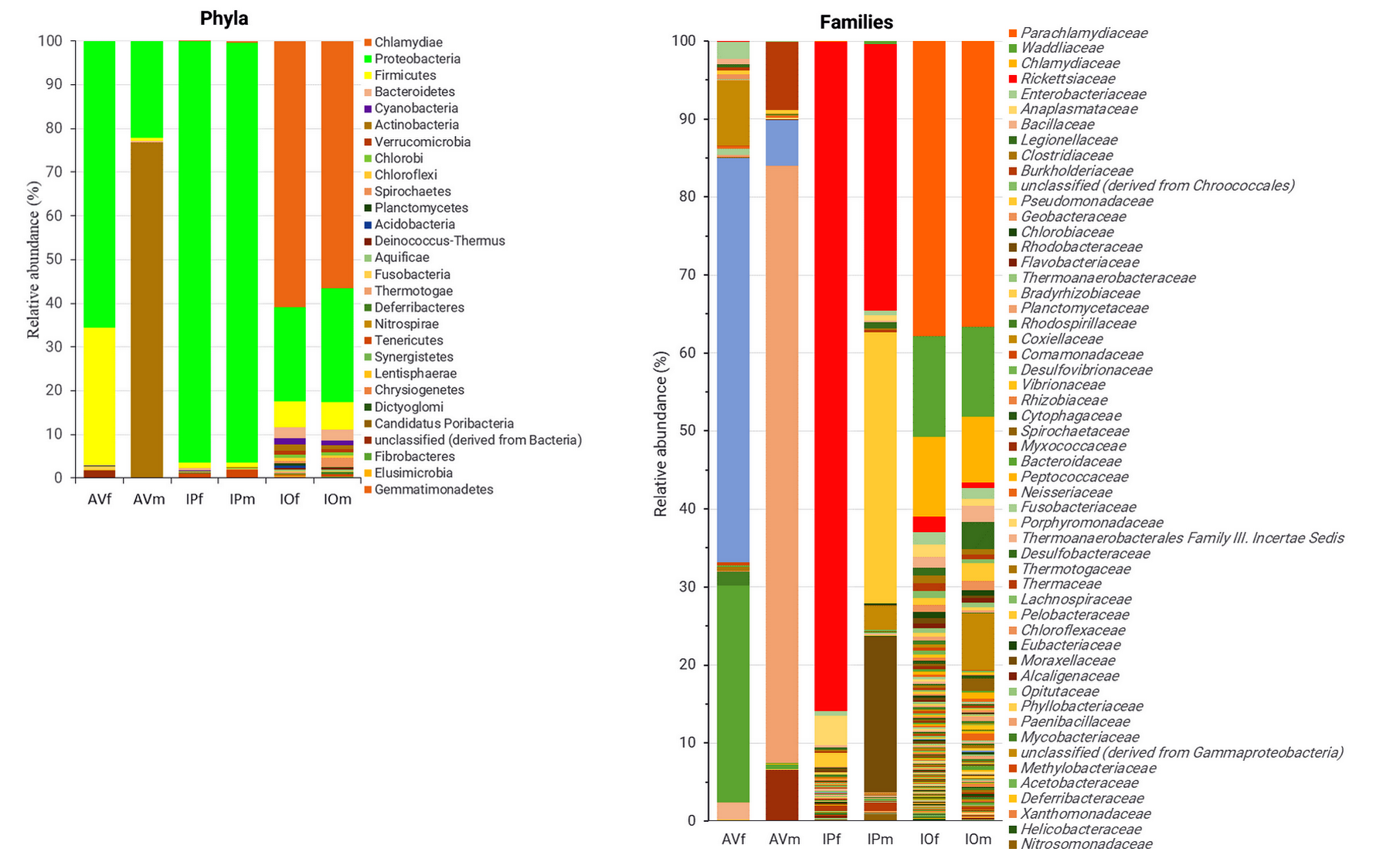

Supplement: Supplementary Figure 2 — Phylum-level and Family-level taxonomic profiles of gut microbiota of AVf, AVm, IPf, IPm, IOf, and IOm. Taxonomical features annotated on MG-TAST, based on RefSeq database at minimal identity cutoff of 60%. Only the most abundant phyla are shown in the legend. [file Image_2.TIF]

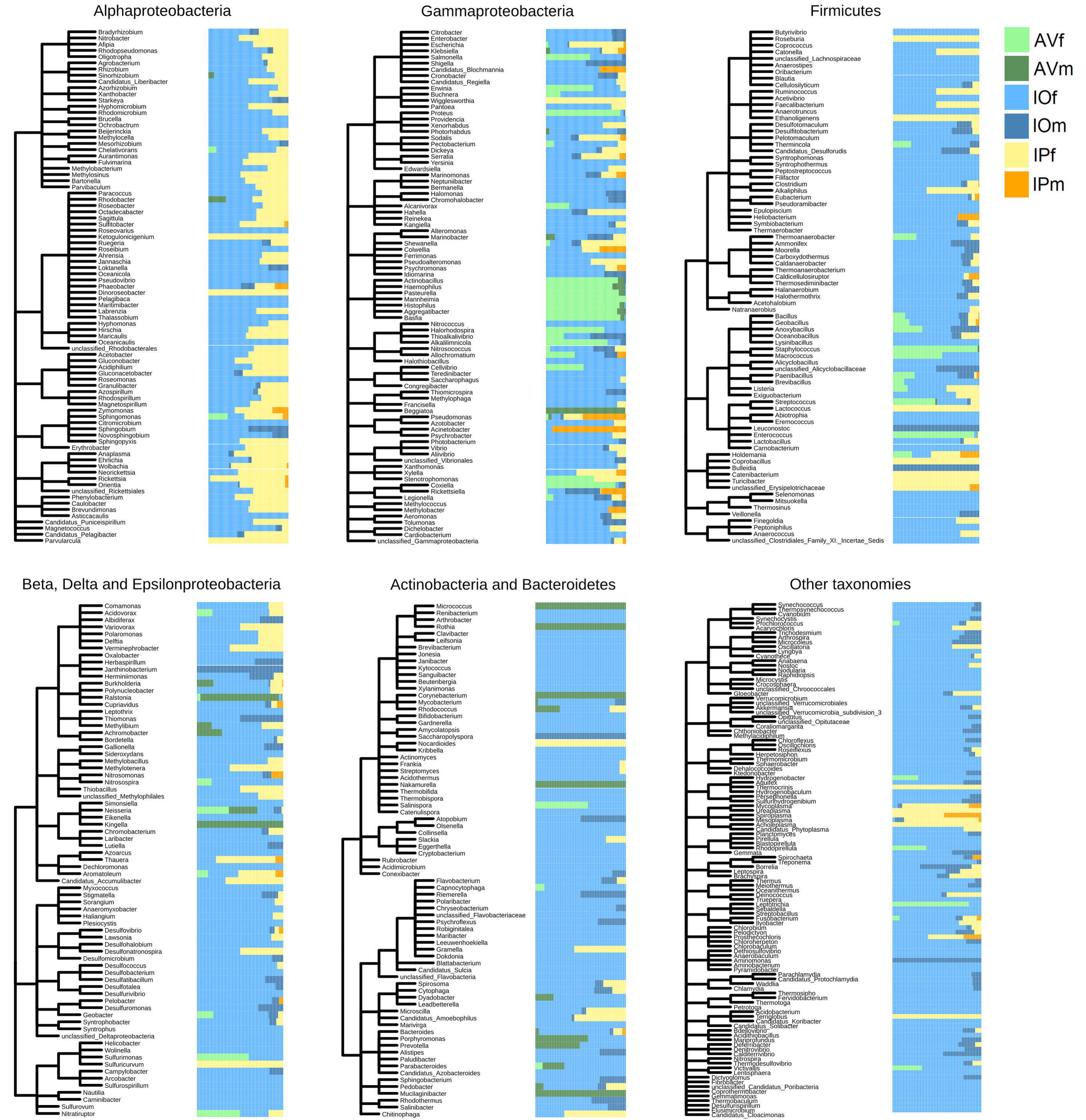

Supplement: Supplementary Figure 3 — Phylogenetic tree and relative abundance of bacterial genera found in the gut microbiota of AVf, AVm, IPf, IPm, IOf, and IOm. The relative abundance of each genus is indicated by the scale bar, and bars are pattern-coded for each tick species and sex. Phylogenetic trees were built using NCBI Taxonomy Browser. For this purpose the taxonomic ID of all genus identified in the taxonomic profiling in MG-RAST pipeline (based on RefSeq database at minimal identity cutoff of 60%) were collected using the Taxonomy name/id tool. The bacteria were then split in six groups according to phylum or class, and the phylogenetic trees were built by submitting the taxonomic ID list of each group to the NCBI Common Tree tool. [file Image_3.TIF]

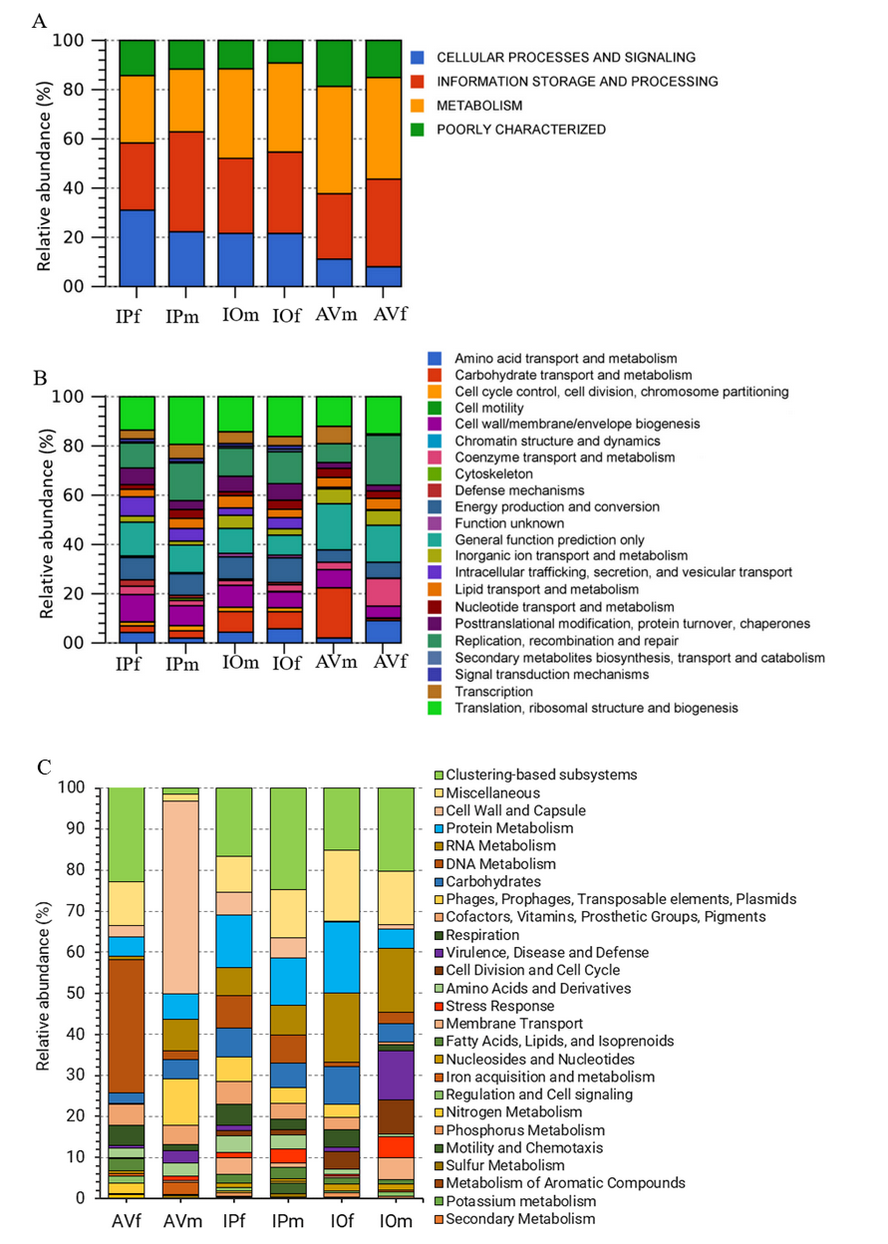

Supplement: Supplementary Figure 4 — The functional profile of AVf, AVm, IPf, IPm, IOf, and IOm based on COG database. (A) Abundance profiles of functions clustered at Level 1 (B) and Level 2. Protein features annotated following the MG-RAST pipeline, profiles acording representative hit method, with maximum e-value cut-off of 1e-5, minimal identity cut off of 80%; and minimum alignment length of 15 bp. (C) The functional profile of gut microbiome of AVf, AVm, IPf, IPm, IOf, and IOm based on the SEED subsystem database at categorical Level 1, with minimal identity cutoff of 80%. [file Image_4.TIF]
